# The Book World of Medicine and Science

**Published:** 1899-09-09

**Authors:** 


### The Book World of Medicine and Science.

A System of Medicine by Many Writers. Edited by Thomas Clifford Allbutt, M.A., M.D., LL.D., F.R.C.P., F.R.S. Vol. VII. (London: Macmillan and Co. 1899. Price 25s.)

The great work which Professor Allbutt set himself is now approaching its fulfilment, and with the volume which is to follow the one which now lies before us the System of Medicine will be complete. It is a work the doing of which must have been a matter of intense interest to its editor, and the full accomplishment of which will, we are sure, be to him a continued source of satisfaction. It can but very seldom happen that so great a book, dealing with such a variety of subjects, and expressing the views of so many minds, contains so very few points in regard to which even the censorious would suggest any alteration. This, the seventh volume, continues the consideration of the diseases of the nervous system, which had been commenced in the volume before. First, there is a good chapter, by Dr. Frederick Taylor, on myelitis, or rather on those forms of myelitis which, not being confined to certain tracts in the cord, may be regarded as diffuse, using the term, "diffuse," not as meaning that the disease is spread throughout a considerable area of the cord, but that it affects all the tissues in the area where it occurs. This disease is treated of under the two heads of acute and chronic—a good clinical distinction. What used to be described as reflex paraplegia is now said to arise from the extension of a neuritis along the nerve trunks and nerve roots. Such are the cases in which the symptoms of myelitis of the lower part of the cord have arisen as a sequel to other diseases, usually of the genito-urinary organs, but sometimes of the intestine.

A good deal of very useful matter is comprised in the portion of this chapter which deals with matters concerning diagnosis. Caisson Disease is written upon by Dr. Andrew Smith, of New York. The article is a good one, but the author does not seem able to throw any new light on the pathology of this very curious condition. Fortunately the treatment of the disease by return to the caisson, or to a compressed air chamber, followed by a very gradual reduction of the pressure, is very successful if undertaken in time. The "limited diseases of the spinal cord" are divided into scleroses and nuclear diseases, a division which is useful enough from a clinical point of view, although we cannot profess much love for classifications founded upon such mixed bases. That one group of diseases end in certain tissue changes and that another set of diseases implicate certain groups of cells, are two separate propositions, neither of which excludes the other. The very complex subject of disseminate sclerosis is very ably dealt with by Dr. Risien Russell, who again and again shows his powers in this volume. He points out how varied may be the symptoms of this condition and how difficult the diagnosis, as, indeed, may well be the case in a malady the lesions of which are scattered in such a random manner throughout the nervous system. Dr. Risien Russell's article makes it very clear that if we are to understand the real meaning of many of the odd nerve phenomena which are occasionally met with almost as isolated symptoms we must cut ourselves adrift from the classical conceptions which have been derived from the study of certain typical cases, and recognise as examples of disseminate sclerosis many cases which might be, and probably have often been, regarded as merely hysterical. "The gravity of an error of diagnosis which would subject a patient with so serious a form of organic disease to the rigid discipline so necessary in the treatment of hysteria is," he says, "very obvious." Tabes dorsalis is dealt with by Dr. Mott and Dr. Ormerod, the latter of whom gives a good picture of its clinical symptoms. Sir T. Grainger Stewart, Dr. Allan Starr, and Dr. Beevor figure largely in the articles in this section, where also appears the only article in this volume by the editor. This short article is upon a subject which will probably attract more attention now that it has been definitely ticketted as a distinct disease, namely, senile paraplegia. Under this heading Professor Allbutt refers to a certain loss of power in the legs which is often to be seen in old people, a loss of power which is not a mere feature of general weakness due to age, but is a true palsy, depending on some local decay of the spinal cord. After falling into a shuffling gait for some months an old man, or woman, may somewhat suddenly find himself unable to walk at all, or may only be able to drag himself along with difficulty. "In my experience," says Professor Allbutt, "many old people are able to walk fairly well until within the space of a few days or weeks they are reduced more or less quickly to a very feeble gait, if not to overt paraplegia. The disability is put down to old age, and regarded as a mere incident in a general failure of power; but the aged person finds himself confined to his chair, while, perhaps, the rest of his faculties may yet for some time be preserved, and suffice for a tranquil enjoyment of life." We fancy that a careful observation of the history of old men will show that this premature and disproportionate failure of leg power is by no means uncommon. There is no doubt that Professor Allbutt is right in regarding it as due to a definite spinal lesion. The section on diseases of the brain opens with three important articles: one on the "Experimental Pathology of the Cerebral Circulation," by Dr. Leonard Hill; one on the "Regional Diagnosis of Cerebral Disease," by Professor Ferrier; and one on "Aphasia and Other Speech Defects," by Dr. Bastian. The latter is a peculiarly attractive essay, and will be read with great interest. The article by Professor Ferrier is full, authoritative, and crammed with information, much of which, however, will, we fancy, not be carried far away from the book. It is a subject which almost demands constant reference to the printed page. Among the most important articles in this section are the two on meningitis, one by Dr. Barlow on "Tuberculous Meningitis," and the other by Dr. Lees and Dr. Barlow on "Simple Meningitis in Children." Together they occupy nearly a hundred pages, and completely cover a most interesting group of diseases which often require the exercise of high diagnostic powers for their elucidation. "Occlusion of Cerebral Vessels" is dealt with by Dr. James Taylor, and "Cerebral Hæmorrhage" by Dr. Howard Tooth, both of whom have written well upon their respective subjects, although rather less fully than might have been expected in such a work. In view of the intimate relation between these two subjects, and the fact that many of the evil effects and of cerebral hæmorrhage are due to the co-incident interference with the distal circulation, it is difficult to see why these two articles should by a curious editorial freak have been separated from each other by the interposition of an essay by Mr. C. A. Balance on "Certain Affections of the Ear," an article which would have found its proper place as an introduction to that of "Abscess of the Brain," by Dr. Byrom Bramwell, which comes further on. "General Paralysis of the Insane" is written about by Dr. Savage and Dr. Goodall, the former dealing principally with diagnosis, prognosis and treatment, in addition to which he gives an interesting page on moral responsibility and testamentary capacity in this disease. Among the "Other Diseases" are articles on "Disorders of Sleep," by Professor Bradbury; "Epilepsy," by Sir William Gowers; "Puerperal Eclampsia," by Dr. Herman; and on Chorea, the Tics, Paramyoclonus Multiplex, Saltatory Spasm, Head-Nodding, &c., by Dr. Risien Russell. Many other articles, which we have not space even to mention, are included in this most interesting volume, which will certainly take its place as the standard book of reference on the subjects which it treats with such authority. We may add that very copious references are given to the various authors quoted.

